# What Is the Potential for Lumacaftor as a Chemical Chaperone in Promoting hERG Trafficking?

**DOI:** 10.3389/fcvm.2022.801927

**Published:** 2022-02-25

**Authors:** Zequn Zheng, Yongfei Song, Jiangfang Lian

**Affiliations:** ^1^Department of Cardiovascular, Medical College, Ningbo University, Ningbo, China; ^2^Ningbo Institute for Medicine & Biomedical Engineering Combined Innovation, Ningbo, China; ^3^Department of Cardiovascular, Lihuili Hospital Affiliated to Ningbo University, Ningbo, China

**Keywords:** hERG channel protein, Lumacaftor, gene mutation, retrafficking, therapeutic potential

## Introduction

Endogenous molecular chaperones are essential in the process of going from nascent chain to folded protein (1). Many studies have confirmed that overexpression of specific molecular chaperones promotes the maturation of corresponding substrate proteins, also known as client proteins, which restores intracellular protein homeostasis and is thought to be an effective treatment in a variety of protein conformational diseases such as cystic fibrosis (CF), Alzheimer's disease (AD), Parkinson's disease (PD), amyotrophic lateral sclerosis (ALS), etc (2). Nevertheless, the rescued effect resulting from endogenous molecular chaperones is not specific, since it may have off-target and even deleterious consequences, such as tumor formation (3).

To date, nearly 500 LQT2-associated pathogenic variants have been identified, of which 40% represent nonsense, frame-shift, or splice-site variants that inhibit hERG protein synthesis (Class 1). The remaining 60% are missense variants causing hERG channel loss of function by either disrupting channel trafficking to the membrane (Class 2, trafficking defective), disruption of channel gating (Class 3), and/or negatively affecting channel conductance (Class 4) (4–7). Functional studies revealed that over 80% of missense mutations cause defective hERG protein trafficking. Therefore, exogenous chemical chaperones generating a pharmacological activity that target specific protein conformation diseases represent the most promising therapeutic options. Lumacaftor (LUM), a medicine licensed by the Food and Drug Administration (FDA) for the clinical treatment of CF, appears to be a good representative of this sort of chemical. In a disease-specific human-induced pluripotent stem cell-derived cardiomyocytes (hiPSC-CMs) model, Mehta et al. first examined its effect on different mutation types of hERG. The results showed that for the two variants of A561V and IVS9-28A>G (Class 2), LUM increases the membrane expression of the protein and corrects the cellular phenotype well, but has no effect on the other two variants representing Class 1 (7). We read with great interest a paper published in the journal of *Circulation-Genomic and Precision Medicine* by O'Hare et al., in which they established that the LUM works on three mutants of the *KCNH2*: G604S, N633S, and R685P. They confirmed that LUM affiliates the mature trafficking of hERG mutant proteins in hiPSC-CMs but not in heterologous TSA201 cells (6). This result is consistent with the previous findings from Mehta et al. published in the *European Heart Journal* (7). More recently, LUM has also been shown to correct the phenotype of two additional missense mutations, A561T and N996I, generated by the gene-editing CRISPR-Cas9 (8). Taken together, these results suggest that LUM not only acts as a chemical chaperone in mutants of CF transmembrane conductance regulator (CFTR) proteins but also has a selective phenotypic correction on certain hERG mutants.

However, this is only an exciting result at the beginning. Through phenotypic analysis, the rescue of the G604S mutation may cause more severe prolongation of the QT interval, which is shown as a prolonged action potential duration at 90% repolarization (APD90) (6). The researchers explained that following LUM, the enhanced trafficking of the mutant hERG channels produced a more pronounced dominant-negative effect. T634S, another hERG point mutation identified as a variant of uncertain significance (VUS), similarly causes intracellular trafficking defects of hERG channel protein that can be rescued by E-4031 rather than LUM (5 μM), indicating that LUM has a selective rescue effect in hERG variants (9). Moreover, LUM did not rescue the *KCNH2*-G601S trafficking mutation in HEK293 cells when administered alone or in combination with novel identified correctors (10, 11).

Obviously, the mutations of *KCNH2* in heterologous systems like HEK293 cells cannot be corrected by LUM, but in hiPSC-CMs, they can. Indeed, autologous disease-specific hiPSC-CMs have irreplaceable advantages over heterologous expression systems or animal models, and the most important of which is that they contain a variety of native cardiac ion channels (12), even if they are still immature, but have well-replicated cardiac electrical activity (13, 14). For the hERG channel, a critical ion channel that functions in the third phase of a cardiac action potential, hiPSC-CMs ensure its relatively accurate gating kinetics and intracellular maturation mechanisms, as well as complete function associated with the auxiliary subunits, β subunits encoded by *KCNE2* (12, 14). Additionally, in the study of O'Hare et al., three hERG variants could not be rescued by LUM in TSA201 cells (data showed only one R685P, and the other two variants were not shown); however, they were all be rescued in the hiPSC-CMs (6). This finding implies that hiPSC-CMs do provide a model advantage for LUM to work in the same mutation context. The model's benefits give us the confidence to conduct clinical translation (15).

The expression of homozygous hERG mutations alone cannot be corrected, whether in homologous or heterologous expression models. Furthermore, there is no indication that chemical chaperones, including LUM, affect the expression of homozygous mutation channels. In O'Hare et al.'s work, however, LUM's rescue of hERG-G604S induced a more serious clinical phenotype, which was attributed to the drug's stronger dominant-negative effect (6). The premise for this explanation is that the three mutants in their investigation are homozygous. When the two alleles are simultaneously mutated and expressed separately, the stable folding effects of LUM generate an approximate wild-type channel, resulting in a longer APD, indicating that the rescue effect of LUM is far less than that of the mutant gene interfering with the corresponding wild-type gene, thus causing deleterious effects. Actually, in their study, this explanation is vague. Since Mehta et al. have demonstrated that LUM affects heterozygous mutations, in this work, almost three variants can be rescued by LUM in hiPSC-CMs (6, 7). Previous studies have shown that when expressed as homozygous, the T634I mutation cannot be corrected, while T634S can, but only E-4031, not LUM, works (9, 16).

**Figure 1:**
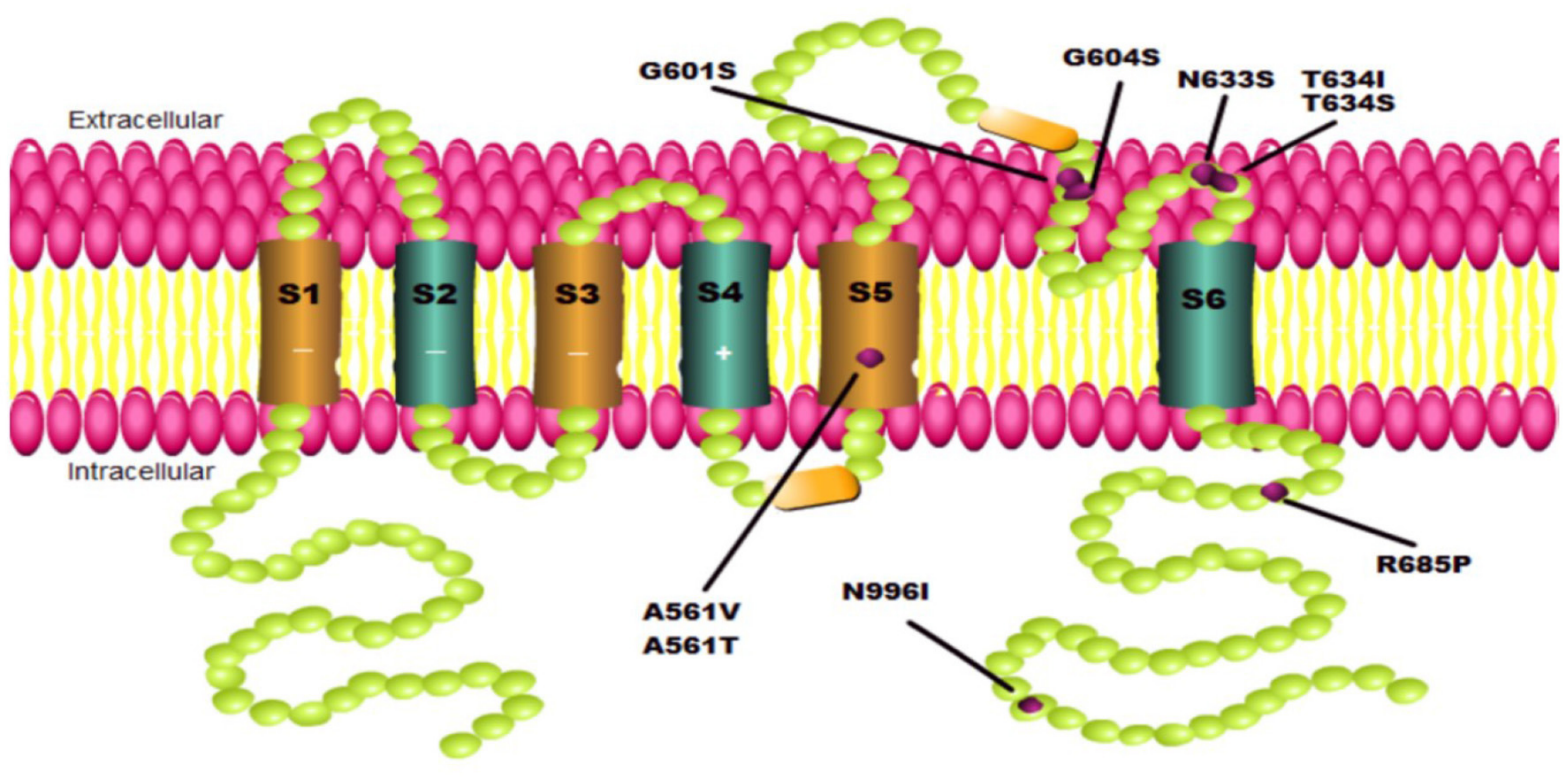
Schematics of *KCNH2* mutations involved in this paper. Each green ball represents an amino acid. Each purple point represents one mutation located on the pore domain to cause a hERG channel trafficking defect.

Interestingly, the conformation changes generated by certain mutations in the hERG channel may differ in the various mutation sites. As a result, LUM's selectivity appears to be confined not only to the greatly near-physiological replication provided by hiPSC-CMs and adverse hERG mutation types but also to specific mutations located at different positions throughout the full-length hERG channel structure. Point mutations in the pore domain of the hERG channel cause a more severe clinical phenotype than those in the N- or C-terminal and other transmembrane segments and are not easy to be corrected, according to functional studies (16). The evidence shows that LUM can correct A561V, G604S, and N633S located in the pore domain but not T634S, which is also in the same location (Figure 1, Table 1). Furthermore, the effect of LUM has not been evaluated for the same mutation location in different cell types. Therefore, attributing this selectivity to the different locations of diverse point mutations in the hERG channel is very difficult. The most plausible explanation is that the conformation of the hERG protein produced by certain individual mutations has such subtle differences that LUM can identify specific mutants while selectively maintaining protein structure. Taken together, the selectivity of LUM may only depend on the exposure of drug-binding sites of protein conformation induced by particular mutations, which is influenced by point mutations at different locations and intracellular environments.

**Table 1:** The effect of the chemical chaperone Lumacaftor on different mutation sites of hERG full-length channel protein.

**Mutations**	**Location**	**Generation**	**Model**	**Effect of Lumacaftor**	**References**
A561V	Pore domain	Disease-specific patients	hiPSC-CMs	Correction of phenotype	(7)
G604S N633S R685P	Pore domain Pore domain C-terminal	Disease-specific patients	hiPSC-CMs	Prolonged APD90 (G604S) Correction of phenotype	(6)
A561T N996I	Pore domain Cytoplasmic tail	CRISPR-Cas9	hiPSC-CMs	Correction of phenotype	(8)
T634S	Pore domain	Transfection	HEK-293	No significant	(9)
T634I	Pore domain	Transfection	HEK-293	No test	(9, 16)
G601S	Pore domain	Transfection	CFBE41o^*^	No significant	(11)

* *CFBE41o, Cystic fibrosis human bronchial epithelial cell line*.

In general, the intracellular mechanism by which LUM promotes the expression of misfolded proteins is unclear. The pharmacological mechanism of LUM has yet to be fully revealed, even in the case of CFTR, and the few studies in LQT2 are much less likely to give a persuasive and comprehensive insight into illuminating its pharmacological mechanism. LUM is generally thought to function primarily through co-translation with folding intermediates (17). As a typical protein conformational disease, mutated CFTR indeed provides a referenceable and available research model for others. In CFTR, indirect evidence suggests that LUM binds directly to the misfolded protein to stabilize its conformation, and then facilitates its escape from the strict cellular protein quality control, such as retention in the endoplasmic reticulum and degradation through the ubiquitin-proteasome system (18). The stability of the conformation enhances the anti-trypsin digestion ability of the full-length channel protein; that is, at the same time, the mutant protein is retained more under the action of LUM (10). Besides, LUM may weaken the main cytoplasmic stress pathways induced by mutation proteins, such as heat shock response (HSR), and thus reduce the expression of related molecular chaperones Hsp70, Hsp90, and others (19). LUM accomplishes this by competing with the most important intracellular protein folding factors, molecular chaperones, and preferentially binding to the misfolded hERG protein. These explanations, however, are only based on circumstantial evidence because there is no visual evidence for the intracellular binding of LUM to hERG proteins, let alone specific binding sites and binding timings. A cryo-electron microscopy structure of CFTR in complex with LUM supports a mechanism in which the correctors stabilize the first transmembrane domain (TMD1) at an early stage of biogenesis, preventing its premature degradation, and thereby allosterically rescuing many disease-causing mutations (20). We expect similar findings of the binding of LUM to hERG-specific domains.

Lumacaftor (LUM) needs to test the consequences of more variants in LQT2 induced by hERG mutations. Additionally, there are still problems in interpreting the result, making the translation from experiment to clinical practice difficult. According to the researchers, using gene-editing tools such as the Dual Integrase Cassette Exchange (DICE) or clustered regularly the interspaced short palindromic repeats (CRISPR)/Cas system in conjunction with high-throughput electrophysiological testing platforms, such as microelectrode array (MEA), to evaluate the role of LUM in multiple single variants of LQT2 may be efficient and beneficial. Understanding what determines the stereo-selectivity of LUM, on the other hand, may be more useful for developing novel specific chemical chaperones and achieving precision medicine. Together, considering the various phenomena presented by LUM, at least the following conclusions can be drawn:

1. Lumacaftor (LUM), as a chaperone for substrate proteins with such a broad stereo-selectivity, could have an impact on other protein conformation disorders.
2. For the same mutation, using two different cell platforms (HEK293 vs. hiPSC-CMs) may lead to different results in terms of translation and transcriptional effects.
3. Lumacaftor (LUM) has been proved to be effective against heterozygous mutations of the *KCNH2* gene. If the prolongation of APD90 of the G604S mutation can be explained by a strong dominant-negative effect, LUM is also effective for homozygous mutations.
4. The intracellular mechanism of LUM's rescue for various mutations in the hERG gene may be involved in the interaction factors during the maturation of hERG protein rather than only depend on mutation specificity.
5. Lumacaftor's (LUM's) selective rescue of different mutants of the same substrate protein reminds us that we should detect the effect of the drug on the hERG mutations as much as possible.

We thank O'Hare et al. for their further contribution to understanding in this regard.

## Author Contributions

ZZ: conceptualization, data curation, writing—original draft, software, and resources. YS: writing—review and editing. JL: conceptualization, writing—review and editing, and supervision. All authors contributed to the article and approved the submitted version.

## Funding

This work was supported by the National Natural Science Foundation of China [Grant Number 81870255] and the Natural Science Foundation of the Zhejiang Province [Grant Number LY21H020001].

## Conflict of Interest

The authors declare that the research was conducted in the absence of any commercial or financial relationships that could be construed as a potential conflict of interest.

## Publisher's Note

All claims expressed in this article are solely those of the authors and do not necessarily represent those of their affiliated organizations, or those of the publisher, the editors and the reviewers. Any product that may be evaluated in this article, or claim that may be made by its manufacturer, is not guaranteed or endorsed by the publisher.
